# Examining the usefulness of the brain network marker program using fMRI for the diagnosis and stratification of major depressive disorder: a non-randomized study protocol

**DOI:** 10.1186/s12888-023-04560-y

**Published:** 2023-01-24

**Authors:** Go Okada, Yuki Sakai, Maki Shibakawa, Toshinori Yoshioka, Eri Itai, Hotaka Shinzato, Osamu Yamamoto, Kenichi Kurata, Tatsuji Tamura, Hiroaki Jitsuiki, Hidehisa Yamashita, Akio Mantani, Norio Yokota, Mitsuo Kawato, Yasumasa Okamoto

**Affiliations:** 1 Department of Psychiatry and Neurosciences, Graduate School of Biomedical and Health Sciences, Hiroshima University, Hiroshima, Japan; 2 Brain Information Communication Research Laboratory Group, Advanced Telecommunications Research Institute International, Kyoto, Japan; 3 XNef, Inc., Kyoto, Japan; 4 Ujina Mental Clinic, Hiroshima, Japan; 5 Kabe Mental Health Clinic, Hiroshima, Japan; 6 Tamura Mental Clinic, Hiroshima, Japan; 7 Jitsuiki Clinic, Hiroshima, Japan; 8 Minna No Suimin Stress Care Clinic, Hiroshima, Japan; 9 Mantani Mental Clinic, Hiroshima, Japan; 10 Yokota Mental Clinic, Hiroshima, Japan

**Keywords:** Magnetic resonance imaging, Depression & mood disorders, Mental health

## Abstract

**Background:**

Although many studies have reported the biological basis of major depressive disorder (MDD), none have been put into practical use. Recently, we developed a generalizable brain network marker for MDD diagnoses (diagnostic marker) across multiple imaging sites using resting-state functional magnetic resonance imaging (rs-fMRI). We have planned this clinical trial to establish evidence for the practical applicability of this diagnostic marker as a medical device. In addition, we have developed generalizable brain network markers for MDD stratification (stratification markers), and the verification of these brain network markers is a secondary endpoint of this study.

**Methods:**

This is a non-randomized, open-label study involving patients with MDD and healthy controls (HCs). We will prospectively acquire rs-fMRI data from 50 patients with MDD and 50 HCs and anterogradely verify whether our diagnostic marker can distinguish between patients with MDD and HCs. Furthermore, we will longitudinally obtain rs-fMRI and clinical data at baseline and 6 weeks later in 80 patients with MDD treated with escitalopram and verify whether it is possible to prospectively distinguish MDD subtypes that are expected to be effectively responsive to escitalopram using our stratification markers.

**Discussion:**

In this study, we will confirm that sufficient accuracy of the diagnostic marker could be reproduced for data from a prospective clinical study. Using longitudinally obtained data, we will also examine whether the “brain network marker for MDD diagnosis” reflects treatment effects in patients with MDD and whether treatment effects can be predicted by “brain network markers for MDD stratification”. Data collected in this study will be extremely important for the clinical application of the brain network markers for MDD diagnosis and stratification.

**Trial registration:**

Japan Registry of Clinical Trials (jRCTs062220063). Registered 12/10/2022.

## Background

Major depressive disorder (MDD) is a psychiatric disorder characterized by a depressed mood, decreased motivation/interest/mental activity, decreased appetite, sleeplessness, and persistent sadness/anxiety. MDD leads to the largest social loss among all illnesses [1, 2]. However, the development of treatments for psychiatric disorders is stagnant, and one of the contributory factors is the limited objective diagnostic methods.

The Diagnostic and Statistical Manual of Mental Disorders (DSM) provides a common language and standard criteria for classifying and diagnosing psychiatric disorders. The diagnosis of MDD is also defined in the DSM. However, the diagnosis depends on the physician’s subjective judgment of clinical symptoms, and the reliability of the diagnosis is considered to be inadequate even with the latest diagnostic criteria, the DSM-5. The concordance rate between testers for MDD diagnosis is very low, with a κ coefficient of 0.28, therefore establishing an objective diagnosis is an urgent issue [3].

Furthermore, MDD diagnosed based on the DSM is not a biologically homogeneous group, and it is considered that different treatments according to the MDD subtypes are required. In fact, it is known that the remission rate for drug therapy is approximately 30% for first-line antidepressants and < 70% even with the use of various treatments [4]. However, no stratification method currently exists to identify a biologically uniform MDD subtype that responds to a particular drug therapy.

Although many studies have reported the biological basis of psychiatric disorders, none have been put into practical use or are useful in clinical practice. Particularly, resting-state functional magnetic resonance imaging (rs-fMRI) is expected to be put into practical use owing to its safety and provision of a large amount of information. However, there is a great difficulty in practical use because the nature of the data differs depending on the measurement facility. Therefore, we integrated rs-fMRI data acquired at multiple institutions by the harmonization method using traveling individuals as homogeneous large-scale data (total of 1,584 cases) [5] and subsequently applied a cutting-edge machine learning method to develop a brain network marker for MDD diagnosis [6]. This brain network marker can distinguish between healthy individuals and patients with MDD with a probability of approximately 70%, even for completely independent data obtained at different facilities [6]. Therefore, we designed this study as a clinical trial to establish anterograde evidence for the practical application of this brain network marker for MDD diagnosis (diagnostic marker) as Software as a Medical Device. In addition, we developed brain network markers for MDD stratification (stratification marker) with robustness among independent datasets by performing unsupervised machine learning on rs-fMRI data of patients with MDD [7]. Statistically significant differences were also observed in the therapeutic responsiveness to escitalopram between the MDD subtypes discriminated by these brain network markers. Therefore, the anterograde verification of these stratification markers was set as the secondary endpoint of this study. The results of this study will provide useful information for the diagnosis and treatment selection of MDD using rs-fMRI.

## Methods/Design

### Study design and setting

This study will be a non-randomized, open-label study involving patients with MDD and healthy controls (HCs).

This trial will be conducted at eight facilities (Hiroshima University Hospital, Kabe Mental Health Clinic, Minna no Suimin Stress Care Clinic, Mantani Mental Clinic, Ujina Mental Clinic, Yokota Mental Clinic, Tamura Mental Clinic, and Jitsuiki Clinic) in Japan.

Three groups of patients with MDD will be enrolled in this study. The first group will continue treatment with escitalopram only for at least the first 6 weeks but will be followed for a total of 6 months without other restrictions on antidepressant treatment (escitalopram group); the second group will receive regular medical care (no restrictions on antidepressant medication) for 6 months and obtain data longitudinally (usual care group); the third group will acquire data only at one point for a cross-sectional study (cross-sectional study group). In addition, for HCs, only one data point will be used. MRI and clinical evaluations in each group will be performed according to the schedule presented in Tables 1, 2 and 3.

**Table 1:** Schedule of escitalopram group and usual care group

Timing Item	Registration date	First measurement date of the MRI	Outpatient examination		
			Consultation day	After 6 weeks	After 6 months
Schedule	-1 week	0 week	N/A^b^	6th week	24th week
Allowance from the specified date	± 1 week	0		± 2 weeks	± 4 weeks
Consent	**○**				
Background information	**○**				
PHQ-9	**○**			**○**	**○**
BDI-II	**○**		**○**	**○**	**○**
DSM-5 (MINI)	**○**				
HRSD		**○**		**○**	**○**
YMRS		**〇**		**〇**	**〇**
SHAPS, PANAS		**○**		**○**	**○**
NEO-FFI, CATS	**○**^a^				
JART, handedness		**○**			
MRI		**○**		**○**	**○**
Medication status	**○**	**○**	**○**	**○**	**○**
Evaluation of adverse events	**○**	**○**	**○**	**○**	**○**

^a^It will be distributed on the day of consent acquisition and can be collected at week 0

^b^No interval is specified to match the schedule of usual medical care

*BDI-II* Beck Depression Inventory-II, *CATS* Child Abuse and Trauma Scale, *DSM-5* Diagnostic and Statistical Manual of Mental Disorders 5th edition, *HRSD* Hamilton Rating Scale for Depression, *JART* Japanese Adult Reading Test, *MINI* Mini-International Neuropsychiatric Interview, *Neo-FFI* Neo-Five-Factor Inventory, *PANAS* Positive and Negative Affect Schedule, *PHQ-9* Patient Health Questionnaire-9, *SHAPS* Snaith-Hamilton Pleasure Scale, *YMRS* Young Mania Rating Scale, *HRSD* Hamilton Rating Scale for Depression

**Table 2:** Schedule of the cross-sectional study group

Timing Item	Registration date	Measurement date of MRI
Schedule	-1 week	0 week
Allowance from the specified date	± 1 week	0
Consent	**○**	
Background information	**○**	
BDI-II	**○**	
DSM-5 (MINI)	**○**	
HRSD		**○**
YMRS		**〇**
CATS	**○**^a^	
handedness		**○**
MRI		**○**
Medication status	**○**	**○**
Evaluation of adverse events	**○**	**○**

^a^It will be distributed on the day of consent acquisition and can be collected at week 0

*BDI-II* Beck Depression Inventory-II, *CATS* Child Abuse and Trauma Scale, *DSM-5* Diagnostic and Statistical Manual of Mental Disorders 5th edition, *MINI* Mini-International Neuropsychiatric Interview, *HRSD* Hamilton Rating Scale for Depression, *MRI* magnetic resonance imaging

**Table 3:** Schedule of healthy controls

Timing Item	Registration measurement date of MRI
Schedule	0 week
Consent	**○**
Background information	**○**
BDI-II	**○**
DSM-5(MINI)	**○**
CATS	**○**
handedness	**○**
MRI	**○**
Evaluation of adverse events	**○**

*BDI-II* Beck Depression Inventory-II, *CATS* Child Abuse and Trauma Scale, *DSM-5* Diagnostic and Statistical Manual of Mental Disorders 5th edition, *MINI* Mini-International Neuropsychiatric Interview, *MRI* magnetic resonance imaging

This trial was registered at jRCT (jRCTs062220063) on 12/10/2022 and will adhere to the Standard Protocol Items: Recommendations for Interventional Trials guidelines [8].

### Purposes of the study

The purposes of this study are as follows:

- Purpose 1. We will verify whether it is possible to distinguish between patients with MDD and HCs using a “diagnostic marker.”
- Purpose 2. We will examine whether this “diagnostic marker” reflects the effects of treatment in patients with MDD.
- Purpose 3. We will longitudinally obtain data on patients with MDD treated with escitalopram and verify whether it is possible to distinguish MDD subtypes that are related to escitalopram treatment response using “stratification markers.”
- Purpose 4. We will examine whether treatment effects can be distinguished by “stratification markers” for drug therapies other than escitalopram.
- Purpose 5. We will use the MRI data obtained in the verification process as learning data for the updated Artificial Intelligence (AI) program that assists in diagnosing MDD for accuracy improvement.

### Primary and secondary endpoints

The primary endpoint will be the area under the curve (AUC) of the receiver operating characteristic (ROC) curve when distinguishing between patients with MDD and HCs using the depression probability value calculated from the diagnostic marker at week 0.

The following will be the secondary endpoints:

1. Longitudinal evaluation of the diagnostic marker: The depression probability value, which is the output of the diagnostic marker, will be analyzed with two factors: remission/non-remission and time of treatment (0 w, 6 w, 6 m). Remission has been defined as a depression severity of 7 points or less using the Hamilton Rating Scale for Depression (HRSD).
2. Depression severity will be obtained longitudinally (0 w, 6 w) in the escitalopram group; we will compare the rate of symptom improvement evaluated using the HRSD among the subtypes identified by the stratification markers in the escitalopram group.
3. Depression severity will be obtained longitudinally (0 w, 6 w) in the usual care group, and we will compare the rate of symptom improvement evaluated using the HRSD among the subtypes identified by the stratification markers in the usual care group.

### Participants

Among the depressed patients who visit the medical facility in this study, those who meet all the inclusion criteria of the patient group (escitalopram, usual care, and cross-sectional study groups) and do not meet any of the exclusion criteria of the patient group will be included in the patient group.

The inclusion criteria for the patient group will be as follows: (1) men and women whose age during consent is between 20 and 75 years, (2) patients diagnosed with MDD following the Mini International Neuropsychiatric Interview (MINI) [9] measured after obtaining consent, and (3) voluntary written consent to participate in this study.

The exclusion criteria of the patient group will be: (1) patients with a mental state in which it is difficult to understand the purpose of the study, (2) patients with MDD with psychotic features, (3) diagnosed with bipolar disorder or schizophrenia, (4) diagnosed with substance abuse within 6 months before obtaining consent, (5) treatment for anxiety disorders within 6 months before obtaining consent, (6) personality disorder coexisting during consent, (7) patients with a marked suicide tendency, (8) patients with MRI contraindications, and (9) patients judged by the principal investigator or the co-investigator to be inappropriate as a research participant.

The HCs will be recruited from local communities. The inclusion criteria of HCs will be (1) men and women aged between 20 and 75 years during consent, (2) persons without any mental illness following MINI measured after obtaining consent, and (3) persons who have voluntarily consented to participate in this study in writing. The exclusion criteria of the HCs will be (1) persons with or with a history of mental or neurological disorders, (2) persons with MRI contraindications, and (3) persons judged by the principal investigator or co-investigator to be inappropriate as a research participant.

### Allocation

Patients with MDD who consent to participate in this study will be assigned to either the escitalopram group, usual care group, or cross-sectional study group based on the following criteria during enrollment. The applicability of each group will be confirmed in the following order: escitalopram group, usual care group, and cross-sectional study group.

The inclusion criteria for the escitalopram group will be as follows: (1) no treatment or less than 2 weeks of antidepressant treatment with an escitalopram-equivalent dose of 5 mg/day or less during the current depressive episode, and (2) patients who are suitable to start treatment with escitalopram based on the clinical judgment of a physician.

The exclusion criteria of the escitalopram group will be as follows: (1) use of mood stabilizers and antipsychotics; (2) receiving electroconvulsive therapy within 3 months before informed consent; (3) receiving standardized psychotherapy (including cognitive-behavioral therapy and interpersonal therapy) other than standard outpatient psychotherapy; and (4) contraindications for escitalopram.

The inclusion criteria of the usual care group will be as follows: (1) no treatment or less than 2 weeks of antidepressant treatment with an escitalopram-equivalent dose of 5 mg/day or less during the current depressive episode; (2) not meeting the inclusion criteria for the escitalopram group or inability to continue escitalopram treatment for 6 weeks.

The exclusion criteria for the usual care group will be as follows: (1) use of mood stabilizers and antipsychotics; (2) receiving electroconvulsive therapy within 3 months before informed consent; and (3) receiving standardized psychotherapy other than standard outpatient psychotherapy.

Regarding the cross-sectional study group, among the patients who meet the participation criteria for this study, those who do not meet the selection criteria for either the escitalopram group or the usual care group are eligible.

### The target number of participants and the basis for setting

The target number of participants in each group is as follows:

- Escitalopram group: 80 participants.
- Usual care group: 60 participants.
- Cross-sectional study group: 200 participants.
- HCs: 50 participants.

The number of cases required for the main examination in this study was calculated as follows:

- The number of participants required to verify that patients with MDD and HCs can be distinguished using a “diagnostic marker” was set based on the following criteria. The AUC was approximately 0.77 in the data of our previous study. [6] Assuming that the AUC is 0.65 conservatively and calculating the sample size (http://www.biosoft.hacettepe.edu.tr/easyROC/) that can detect AUC > 0.5 with Type, I error: 0.05 and power: 0.8, 43 participants will be required for each group. Assuming a 10% chance of cancellation or dropout, it was considered appropriate to aim for 50 registrations per group.
- The reasons for setting the number of cases required to verify whether the rate of symptom improvement among the MDD subtypes identified by the stratification markers in the escitalopram group differed significantly are as follows. Based on a power analysis for the Mann–Whitney U test using the method developed by Shieh et al. [10], the sample size calculation was performed with Type I error 0.05, power: 0.8 using R’s shiehpow function (https://www.rdocumentation.org/packages/wmwpow/versions/0.1.3/topics/shiehpow). Therefore, 68 participants will be required. Predicting approximately 10% cancellation or dropout, it was considered appropriate to aim for the registration of 80 participants.
- Based on past medical records, approximately 60 and 200 patients are expected to be eligible for the usual care group and cross-sectional study group within the registration period, respectively. The data obtained from the escitalopram and usual care groups will be used to determine whether the “brain network marker of MDD diagnosis” reflects the therapeutic effects in patients with depression (Purpose 2). In addition, we will conduct exploratory investigations to determine whether there is a significant difference in the rate of symptom improvement between subtypes by “stratification markers” in the data of the usual care group combined with the data that XNef, Inc. possesses (Purpose 4). Furthermore, the MRI data obtained from all groups will be used as training data to improve the accuracy of the AI program that assists in diagnosing MDD (Purpose 5). Based on these findings, obtaining as much data as possible from eligible patients in non-interventional groups is desirable. Therefore, although it is difficult to imagine a situation where the expected number of cases is significantly exceeded in reality, if the expected number of cases is exceeded, all patients who have provided consent within the registration period should be allowed to be enrolled.

### MRI acquisition

MRI scans will be obtained for all participants using a 3.0 Tesla MRI system (Siemens MAGNETOM Skyrafit; Siemens, Erlangen, Germany) at Hiroshima University. Additionally, rs-fMRI scans will be acquired for 10 min from each participant using the following scanning parameters: slice number, 40; matrix size, 64 × 64; FOV, 212 mm; voxel size, 3.3 × 3.3 × 3.2 mm (slice gap, 0.8 mm); TR, 2,500 ms; TE, 30 ms; flip angle, 80°. T1-weighted structural images will also be acquired using the following scanning parameters: matrix size, 320 × 300 × 224; FOV, 256 × 240 × 179.2 mm; voxel size, 0.8 × 0.8 × 0.8 mm.

### Preprocessing and calculation of the resting-state FC matrix

The preprocessing and calculation of the resting-state FC matrix have been described in detail elsewhere. [6] We will preprocess the rs-fMRI data using FMRIPREP version 1.3.2. [11] The first 10 s of the data will be discarded to allow T1 equilibration. The preprocessing steps include slice-timing correction, realignment, coregistration, distortion correction using a field map, segmentation of T1-weighted structural images, normalization to the Montreal Neurological Institute space, and spatial smoothing with an isotropic Gaussian kernel of 6 mm full-width at half maximum. To analyze the data, we will use the ciftify toolbox version 2.3.2. [12] We will use Glasser’s 379 surface-based parcellations (cortical 360 parcellations + subcortical 19 parcellations) as regions of interest (ROIs); [13] the blood oxygen level-dependent (BOLD) signal time courses will be extracted from these 379 ROIs. A temporal bandpass filter will be applied to the time series using a first-order Butterworth filter with a pass band between 0.01 and 0.08 Hz. Framewise displacement will be calculated for each functional session, and we will remove volumes with a framewise displacement > 0.5 mm along with the previous and two subsequent volumes. [14] If the ratio of the excluded volumes after scrubbing exceeds 47%, the participants will be excluded from the primary analysis. [6] FC will be calculated as the temporal correlation of rs-fMRI BOLD signals across 379 ROIs for each participant. Fisher’s *z*-transformed Pearson’s correlation coefficients will be calculated between the preprocessed BOLD signal time courses of each possible pair of ROIs and used to construct 379 × 379 symmetrical connectivity matrices. We will use 71,631 FC values ([379 × 378] / 2) of the lower triangular matrix of the connectivity matrix for further analysis.

### Analysis of the primary endpoint

As 100 classifiers of MDD (tenfold cross-validation × 10 subsamples) were created in the original study [6], we will apply all of these classifiers to the new dataset and average the 100 outputs (depression probability value) for each participant.

A ROC curve will be estimated for discrimination between HCs and patient groups (regardless of the escitalopram group, usual care group, or cross-sectional study group, the 50th or less in the order of registration will be analyzed) based on the depression probability value. We will display the (sensitivity, 1-specificity) coordinate position corresponding to the optimal cutoff value based on the Youden index, along with point estimates of AUC, optimal cutoff value, sensitivity, and specificity. For AUC, if a 90% confidence interval is constructed and the lower endpoint of the confidence interval is ˃ 0.5, it will be interpreted as AUC > 0.5, with a type I error of 5%.

### Analysis of secondary endpoints

The patients will be grouped into remission and non-remission groups based on the severity of depression at 6 weeks and 6 months, using the escitalopram and usual care groups as the populations to be analyzed. The depression probability value will be analyzed for two factors using these data: remission/non-remission *treatment time point (0 w, 6 w, 6 m), and a test will be conducted at a significance level of 5%.

With escitalopram as the target group for analysis, patients with MDD will be stratified using stratification markers. The rate of symptom improvement evaluated using the HRSD will be compared between depression subtypes and tested at a significance level of 5%.

Patients with MDD in the usual care group combined with the data from XNef, Inc. will also be stratified using the stratification markers. The rate of symptom improvement evaluated using HRSD will be compared between depression subtypes and tested at a significance level of 5%.

### Monitoring and auditing

A person in charge of monitoring will be appointed, and monitoring will be conducted to confirm whether this research is being conducted safely and per the study protocol and whether data are being collected accurately.

When investigators become aware of the occurrence of a disease or malfunction, they will immediately take appropriate measures, such as discontinuing the intervention and entering the information into the Electronic Data Capture without discrepancies.

Data management, statistical analysis, monitoring, and auditing will be outsourced to SRD Co. Ltd.

To ensure the quality of clinical research, the auditor will confirm that it is being conducted appropriately by directly viewing the source documents.

### Patient and public involvement

No patient was involved.

## Dissemination

The findings from this study will be published in peer-reviewed journals and presented at local, national, and international conferences. In addition, the main results will be published on the jRCT website (https://jrct.niph.go.jp/). Hiroshima University and XNef, Inc. will be credited with disseminating both preliminary and final results.

## Discussion

Given fMRI can non-invasively evaluate changes in brain activity with a high spatial and temporal resolution, it has been applied to research on psychiatric disorders, and many results have been reported. For clinical applications, rs-fMRI is attracting attention as a method that can evaluate brain activity without frequently difficult tasks in patients with cognitive and motivational deficits. Initially, analyses were performed to detect statistically significant differences by comparing patients and HCs; however, recently, machine learning methods aimed at discrimination at the individual level have been used. Meta-analyses [15, 16] of such studies have shown good discriminative results, raising expectations for clinical applications. However, it has been highlighted that when commonly used machine learning methods are applied to a small data sample of dozens of individuals, overfitting causes inflation in leave-one-out cross-validation discriminant results, resulting in poor generalization performance to independent external data [17]. Our diagnostic marker [6], which was developed by integrating fMRI data acquired at different facilities as large-scale data by harmonization using traveling individuals [5], has a generalization performance to multiple external independent data with an accuracy of approximately 70%. Therefore, it is considered suitable for prospective clinical research aimed at practical applications. In this study, we will confirm that sufficient accuracy of the diagnostic marker [6] could be reproduced for data from a prospective clinical study.

The diagnostic marker developed by supervised machine learning, which treats DSM diagnosis as the correct answer, cannot address the heterogeneity of MDD. As recently proposed [18], it is necessary to classify patients into homogeneous subtypes from the viewpoint of brain function using unsupervised machine learning methods, including clustering without diagnostic labels, and subsequently evaluate the validity of the classification based on outcomes, such as response to specific treatments. Based on this idea, we previously used the Bayesian multiple co-clustering methods to analyze multidimensional data of patients with MDD and reported that functional connectivity of the brain centered in the right angular gyrus and childhood traumatic experiences categorized the patients into three groups, one of which was less responsive to selective serotonin reuptake inhibitor treatment [19]. However, in addition to the fact that the study was conducted on a relatively small sample size and the reproducibility was not verified, there were limitations, such as using psychological experiences for classification. A study by Drysdale et al., using large multicenter rs-fMRI data, classified patients with MDD into four subtypes according to the functional connectivity patterns of the limbic and frontostriatal networks. Furthermore, the subtypes differed in their responsiveness to transcranial magnetic stimulation therapy. However, a study reproducing the analysis pipeline of Drysdale et al. and analyzing different clinical data sets failed to validate subtype stability [20]. Therefore, the development of reproducible stratification biomarkers is of paramount importance. Recently, we successfully created generalizable stratification markers [7]. However, in the current diagnosis and treatment of MDD, no objective biomarker exists, and treatment is performed by trial and error based on the experience of doctors. Our brain network markers for MDD diagnosis and stratification can overcome this situation. We can provide advanced diagnostics and treatments by verifying and putting them into practical use.

This study design has some limitations, including that our imaging protocol developed in the Strategic Research Program for Brain Sciences (November 2013 to March 2018) has a lower signal-to-noise ratio and resolution than the latest imaging protocols, such as the Harmonization Protocol (HARP) [21]. However, multicenter data collection has just begun regarding the HARP protocol, and there is still no evidence for creating generalizable biomarkers for psychiatric disorders. Therefore, it is necessary to aim for the clinical application of a protocol that already has evidence. Although with some limitations, the data collected in this study will be crucial for the clinical application of brain network markers for MDD diagnosis and stratification.

## Data Availability

Not applicable.

## Abbreviations

MDD: Major depressive disorder
DSM: Statistical Manual of Mental Disorders
rs-fMRI: Resting-state functional magnetic resonance imaging
HCs: Healthy controls
AUC: Area under the curve
ROC: Receiver operating characteristic
HRSD: Hamilton Rating Scale for Depression
MINI: Mini International Neuropsychiatric Interview
ROI: Region of interest
BOLD: Blood oxygen level-dependent
HARP: Harmonization Protocol
